# Development and Vertical Tests of CEPC 650-MHz Single-Cell Cavities with High Gradient

**DOI:** 10.3390/ma14247654

**Published:** 2021-12-12

**Authors:** Song Jin, Peng Sha, Weimin Pan, Jiyuan Zhai, Zhenghui Mi, Feisi He, Chao Dong, Lingxi Ye, Xiangcong He

**Affiliations:** 1 Accelerator Division, Institute of High Energy Physics, Chinese Academy of Sciences, Beijing 100049, China; jinsong@ihep.ac.cn (S.J.); zhaijy@ihep.ac.cn (J.Z.); mizh@ihep.ac.cn (Z.M.); hefs@ihep.ac.cn (F.H.); dongchao@ihep.ac.cn (C.D.); yelx@ihep.ac.cn (L.Y.); hexiangcong@ihep.ac.cn (X.H.); 2 Key Laboratory of Particle Acceleration Physics & Technology, Institute of High Energy Physics, Chinese Academy of Sciences, Beijing 100049, China; 3 Center for Superconducting RF and Cryogenics, Institute of High Energy Physics, Chinese Academy of Sciences, Beijing 100049, China; 4 University of Chinese Academy of Sciences, Beijing 100049, China

**Keywords:** SRF cavity, electro-polishing, quality factor, accelerating gradient, vertical test, buffered chemical polishing

## Abstract

A circular electron positron collider (CEPC) will adopt hundreds of 650-MHz superconducting cavities with high quality factor (*Q*) and accelerating gradient (*E*_acc_). Two 650-MHz single-cell cavities made of fine-grain niobium were first treated via buffered chemical polishing (BCP), which was easy and convenient. However, the vertical test results could not meet the specification of the CEPC (4 × 10^10^ at 22 MV/m). Therefore, electro-polishing (EP) of 650-MHz single-cell cavities was conducted, which was complicated but remarkably effective. Both 650-MHz single-cell cavities achieved state-of-the-art gradients of 35 MV/m after the EP process, which is extremely high for large elliptical cavities (frequency < 1 GHz). One cavity achieved an intrinsic quality factor (*Q*_0_) of 4.5 × 10^10^ at 22.0 MV/m, which was higher than the CEPC spec. The other cavity obtained a lower *Q*_0_ of 3.4 × 10^10^ at 22.0 MV/m, which may have resulted from the cancellation of high-temperature annealing.

## 1. Introduction

Superconducting radio frequency (SRF) cavities made of niobium with a high quality factor and an accelerating gradient are widely adopted for large accelerators worldwide [1]. SRF cavities of different frequencies and shapes have been developed for different kinds of accelerators [2,3,4]. Among these cavities, large elliptical cavities (frequency < 1 GHz) account for a substantial proportion, and are used to accelerate electrons or protons in synchrotron sources (Taiwan Photon Source, High Energy Photon Source, Shanghai Synchrotron Radiation Facility) [5], colliders (KEK B-Factory, Beijing Electron Positron Collider II, Future Circular Collider) [6,7], and proton/neutron sources (Proton Improvement Plan-II, Spallation Neutron Source, European Spallation Source, China Spallation Neutron Source Upgrade, China Initiative Accelerator Driven System) [8,9,10,11]. Compared with 1.3-GHz cavities [12,13,14,15], the global development of large elliptical cavities is relatively slow. There are still technical challengers, such as high *Q* and gradient, field emission, etc.

**Figure 1:**
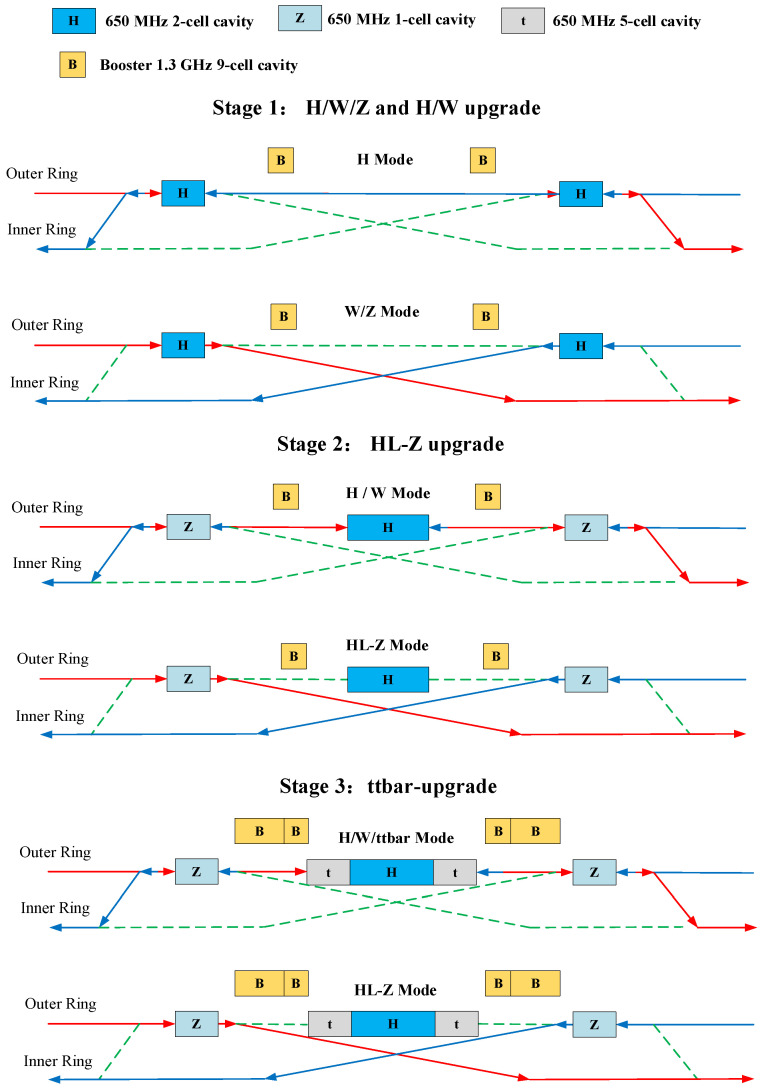
The new scheme of the CEPC SRF system.

The CEPC is a high-energy collider as the Higgs factory, which can also operate in W and Z modes [16,17]. In the CEPC CDR, 650-MHz two-cell cavities are adopted for the collider ring, while 1.3-GHz nine-cell cavities are used for the booster ring. All these cavities are fully installed at one time, which reduce the costs of SRF and cryogenics. However, the luminosity of the Z pole is limited by the impedance and the higher order mode power of the 650-MHz two-cell cavities in the collider ring [18]. Therefore, a new scheme for the SRF system is proposed for a high-luminosity Z (HL-Z) [19], which is shown in Figure 1. The electron beam moves forward along the solid blue lines, while the positron beam moves along the solid red lines. The dashed green lines mean a use in other modes. In the new layout, 650-MHz single-cell and five-cell cavities are added for stage 2 and stage 3. During operation in the HL-Z mode, one hundred and twenty 650-MHz single-cell cavities are used.

The 650-MHz cavities in the collider ring of the CEPC operate in the CW mode, with the spec of *Q*_0_ > 4 × 10^10^ at 22 MV/m (at 2.0 K) during the vertical test (VT). The previous values of *Q*_0_ for large elliptical cavities are much lower than those of the CEPC spec. The European Spallation Source (ESS) adopted the 704.42-MHz β = 0.86 cavities with the spec of 5.0 × 10^9^ at 19.9 MV/m [20], while the Spallation Neutron Source (SNS) used 805-MHz β = 0.81 cavities with the spec of 5.0 × 10^9^ at 15.8 MV/m [8]. However, the 650-MHz β = 0.92 cavity of the Proton Improvement Plan-II (PIP-II) requires *Q*_0_ of 3.0 × 10^10^ at 18.8 MV/m [10], which is clearly higher than those of SNS and ESS. Hence, the spec of the CEPC 650-MHz cavities is challenging compared to other similar cavities.

At present, BCP and EP are two kinds of treatments that obtain high *Q*_0_ and *E*_acc_ for large elliptical cavities [10,21]. BCP is easy and convenient, but EP can achieve a higher gradient. The goal of this article is to figure out which treatment is more feasible to reach the CEPC VT spec. Two 650-MHz single-cell β = 1 prototype cavities (650S4, 650S5) for the CEPC were fabricated, processed using BCP and EP, and tested, and one of them finally exceeded the CEPC VT spec.

## 2. Detailed Treatments of 650-MHz Cavities

### 2.1. BCP Process of 650-MHz Cavities

**Figure 2:**
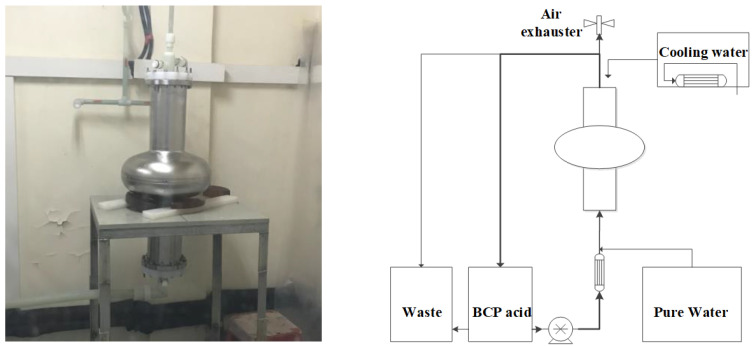
BCP process of 650 MHz single-cell cavity (**left**: setup; **right**: schematic).

The main RF parameters of the 650-MHz single-cell cavities are shown in Table 1; both are made of fine-grain niobium. *B*_peak_ denotes the peak surface magnetic field of the cavity, while *E*_peak_ denotes the peak surface electric field. After fabrication, the 650-MHz cavities first received post processing based on BCP, because there was no EP facility for the 650-MHz cavity at that time. The setup and schematic of the BCP process is shown in Figure 2. A mixture of hydrofluoric acid (HF, 40%), nitric acid (HNO_3_, 69.5%), and phosphoric acid (H_3_PO_4_, 85%) at a ratio of 1:1:2 was injected from the bottom flange of the cavity, and outflowed from the top flange. The acid mixture was circulated in the 650-MHz single-cell cavity at a temperature of 15 °C. Compared with EP, BCP is much easier and cheaper, which is the preferred alternative for the mass cavities of large SRF accelerators.

Both the 650-MHz single-cell cavities (650S4, 650S5) were subjected to the same process: bulk BCP (~200 μm), annealing at 800 °C for 3 h, and light BCP (~30 μm). The processes above were carried out at Ningxia Orient Superconductor Technology Co., Ltd. (Ningxia, China) (OSTEC) in the city of Shizuishan. Afterwards, the 650-MHz single-cell cavities were transferred to Beijing, received a high-pressure rinse (HPR) and were assembled with flanges in a cleanroom. Finally, 650S4 and 650S5 were baked at a low-temperature at 120 °C for 48 h.

### 2.2. Vertical Test of 650-MHz Cavities BCP Processed

**Figure 3:**
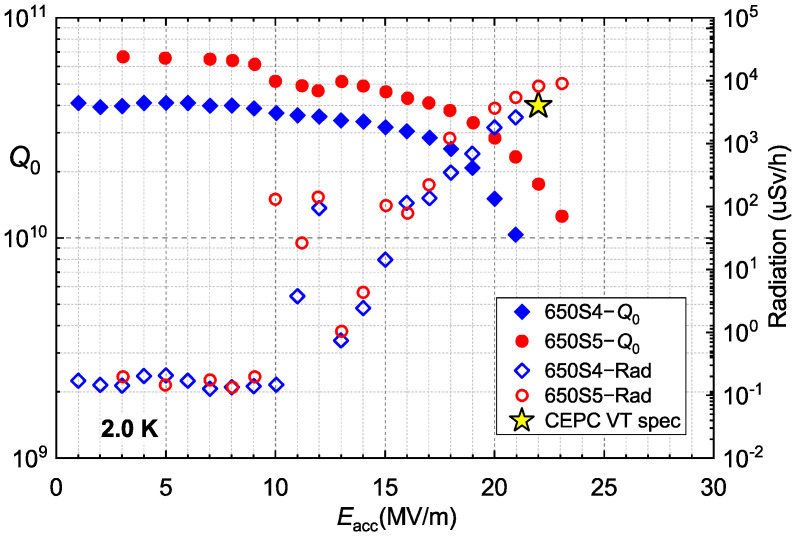
Vertical test results of the 650-MHz single-cell cavities that were processed with BCP.

The vertical test results (BCP baseline) of the 650-MHz single-cell cavities are shown in Figure 3. 650S4 quenched at 21.0 MV/m with *Q*_0_ of 1.0 × 10^10^ and the *Q*_0_ of 650S5 was 4.0 × 10^10^ at 17.0 MV/m, which was higher than that of 650S4. 650S5 quenched at 23.1 MV/m with *Q*_0_ of 1.3 × 10^10^. There was an obvious field emission for both the 650-MHz cavities during the vertical test, which occurred above 10 MV/m. This may have been induced by contamination during assembly in the cleanroom or during transfer. The phenomenon of the *Q*-slope is evident at the medium field, which is common for fine-grain cavities processed with BCP [7,22,23]. In brief, the 650-MHz single-cell cavities could not reach the CEPC VT spec (*Q*_0_ = 4 × 10^10^ at 22 MV/m) with the BCP process.

### 2.3. Setup of the EP Facility

**Figure 4:**
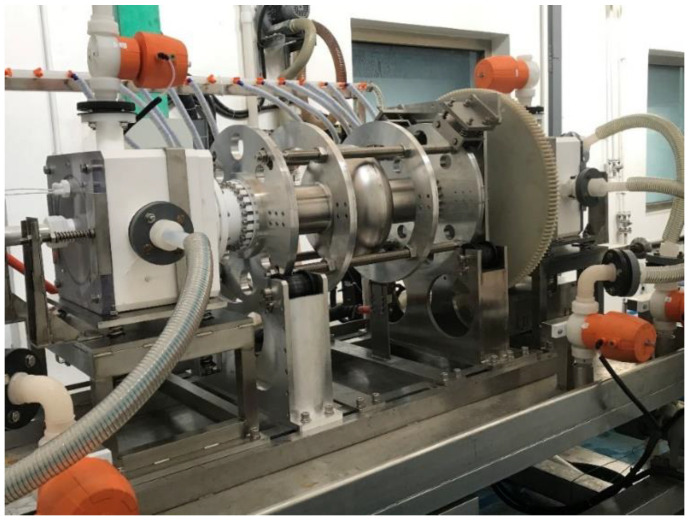

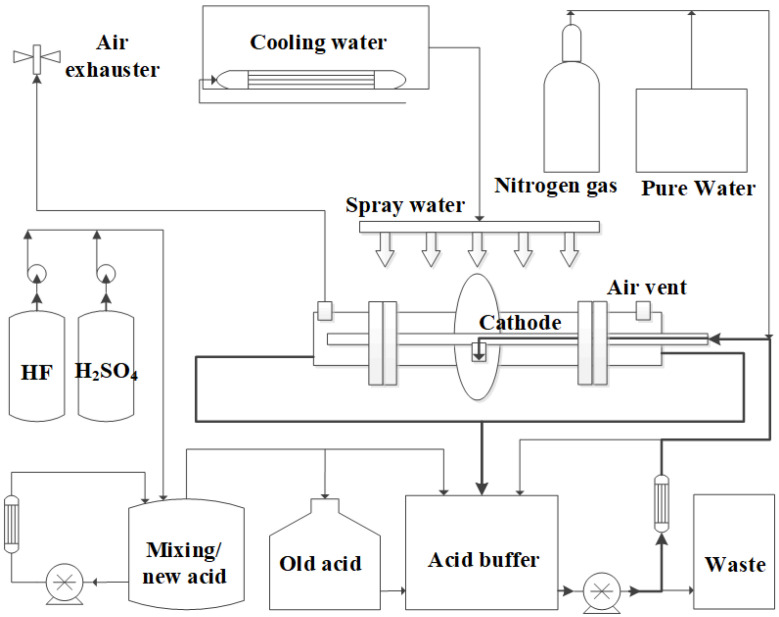
EP process of the 650-MHz single-cell cavity. (**Top**: setup; **bottom**: schematic diagram of the EP piping system for the 650-MHz single-cell cavity).

Because BCP treatment could not reach the CEPC VT spec, a new EP facility was then developed. EP technology, which was originally developed by Siemens [24] and later widely adopted for processing of SRF cavities [25,26,27], has proved that it can significantly improve cavity performance. The EP process of the 650-MHz single-cell cavity was conducted at the new EP facility, which was also used for 1.3-GHz single-cell and nine-cell cavities [28]. The mechanical part was upgraded to fit the dimensions of the 650-MHz cavity, which had a bigger beam tube and equator than those of the 1.3-GHz cavity. The EP process for the 650-MHz single-cell cavity is shown in Figure 4.

The electrolyte used was made of H_2_SO_4_ and HF with a concentration of 96% and 40%, respectively, at a ratio of 9:1. The electrolyte mixing unit mainly consisted of two acid containers and a mixing tank. Because of the heat generated in the process of acid mixing, a cooling system consisting of a heat exchanger and a chiller was also developed for the mixing tank. An acid storage tank and a buffer tank were adopted for EP circulation in the facility. Old acid was stored in an acid storage tank and fresh electrolyte stored in the mixing tank is used for the final EP. A hollow tubular aluminum cathode was inserted along the cavity axis, which injected the electrolyte to the cavity. There was a great deal of hydrogen created during the EP process; thus, the level of electrolyte in the cavity was kept at about 60% to avoid excessive hydrogen bubbles generated at the cavity surface. There was also an exhaust fan to pump out hydrogen, while redundant air was injected through a filter at the other end of the cavity to dilute the hydrogen concentration. During the EP process, heat on the scale of several kilowatts was generated, which is harmful to a cavity. Thus, it is key to control the temperature of the cavity and the acid. Two methods were adopted. First, the electrolyte was cooled through a heat exchanger and a chiller. Second, the outer surface of the cavity was rinsed with cooling water. Finally, the electrolyte was pumped back into the buffer tank when the EP was completed. In brief, the EP process is much more complicated than BCP.

### 2.4. EP Optimization for 650-MHz Single-Cell Cavities

Prior to formal EP of the 650-MHz single-cell cavities, the electrolyte tank and pipes were flushed with sulfuric acid (96%) to eliminate any possible water left in the system. Experiments were carried out to obtain the optimal parameters of EP. A continuous-current oscillation mode was adopted for the EP process. Current oscillation was observed with an in situ QA/QC indicator. The main parameters of the acid flow rate, polishing voltage, acid temperature, and cavity body temperature were monitored and controlled. After the voltage was turned off, the procedure of continuing acid flow along with cavity rotation was adopted to inhibit possible niobium oxide, which might affect cavity performance [29]. Then, a post-EP cleaning procedure was done, including in situ water rinsing, ultrasonic cleaning with detergent, and HPR.

**Figure 5:**
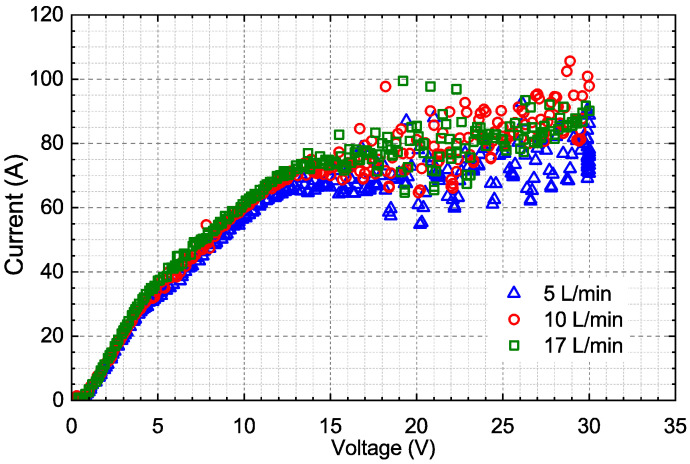
Effects of flow rates on I-V characteristics of EP for the 650-MHz single-cell cavity.

The effects of the flow rates on the current–voltage (I-V) characteristics are shown in Figure 5. The I-V characteristics were similar, along with the increase in the flow rate from 5 L/min to 17 L/min. The current of 17 L/min was slightly higher than that of 5 L/min. Therefore, it was considered that the flow rate slightly affected the I-V characteristics of EP for the 650-MHz single-cell cavity. It was thought that a temperature of EP of less than 20 °C would be low enough for high-gradient cavities [25]. Therefore, 17.5 °C was adopted as our conventional EP temperature in consideration of the efficiency and convenience of the operation, which had been successfully attempted for 1.3-GHz nine-cell cavities [28].

**Figure 6:**
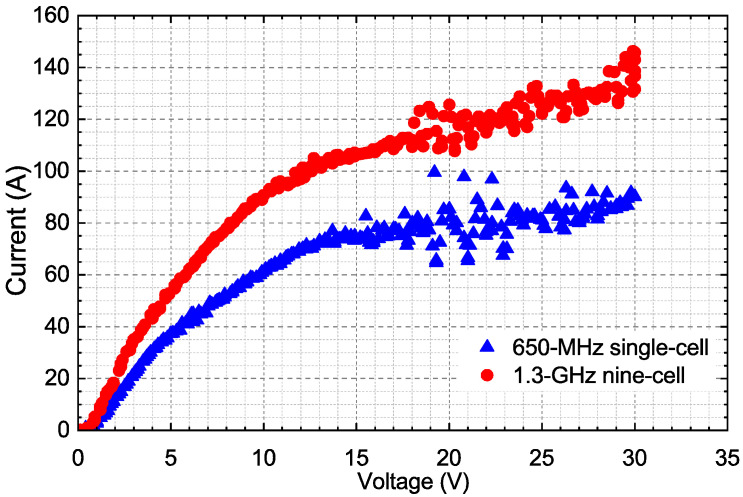
Typical I-V characteristics of EP for 650-MHz single-cell and 1.3-GHz nine-cell cavities.

The typical I-V characteristic curves of EP for the 650-MHz single cell and the 1.3-GHz nine cells are compared in Figure 6; the temperatures of the acid and the cavity body were ~17.5 °C, and the flow rates were ~15 L/min. The increase in current was not linear with voltage for both I-V characteristics. There were inflection points, shown in Figure 6, which approximately divided each I-V curve into two regions: an etching region and a polishing region. The inflection points were about 10 V for the 1.3-GHz nine-cell and 13 V for the 650-MHz single-cell cavities, respectively. This might also result from the large diameter of the 650-MHz single-cell cavity, which was almost two times that of the 1.3-GHz nine-cell cavity. The beam tube of the 650-MHz single-cell cavity restricted the diameter of the cathode to keep the same ratio of the two distances from cathode to beam tube and to equator as 1.3-GHz nine-cell cavity. Hence, the conventional voltage of 17–18 V for the 1.3-GHz nine-cell cavity was abandoned for the 650-MHz single-cell cavity; instead, a voltage of 21 V was adopted with a range of 19–23 V.

### 2.5. EP Process of 650S4 and 650S5

First, 650S5 was subjected to the EP process with a removal of 215 μm, which was used for commissioning of the EP. The temperature was always below 25 °C during this process, which restrained the absorption of hydrogen by the niobium cavity. Then, another removal of ~100 μm was conducted at an optimized working point of 21 V with a temperature around 17.5 °C and a flow rate of ~15 L/min to reset the cavity surface. In addition, high-temperature annealing was omitted, which is commonly used for hydrogen degassing. The effects were evaluated using the vertical test results. The detailed process of 650S5 is as follows:

- EP machine commissioning (~215 μm at <25 °C for both acid and cavity body);
- Reset the cavity surface by EP (~100 μm at ~17.5 °C for both acid and cavity body).

Secondly, 650S4 received a two-step EP process with a voltage of 21 V and a flow rate of ~15 L/min. Because 650S4 previously received bulk BCP and we wanted to determine the synergetic effect of bulk BCP + light EP, so light EP was adopted instead of bulk EP (~150 μm) prior to high-temperature annealing. The detailed process of 650S4 is listed as follows:

- Light EP (~20 μm at ~25 °C for both acid and cavity body);
- High-temperature annealing (950 °C for 3 h);
- Light EP (~20 μm at ~17.5 °C for both acid and cavity body).

### 2.6. Inspection after EP Process

**Figure 7:**
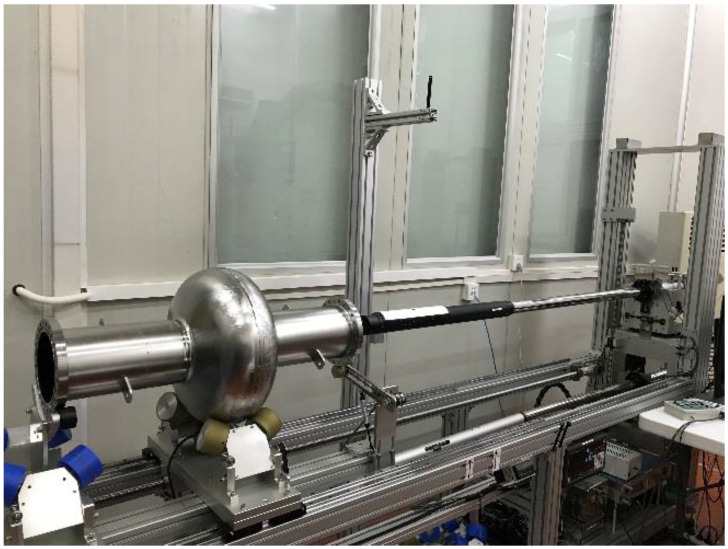
The inspection of the 650-MHz single-cell cavity.

**Figure 8:**
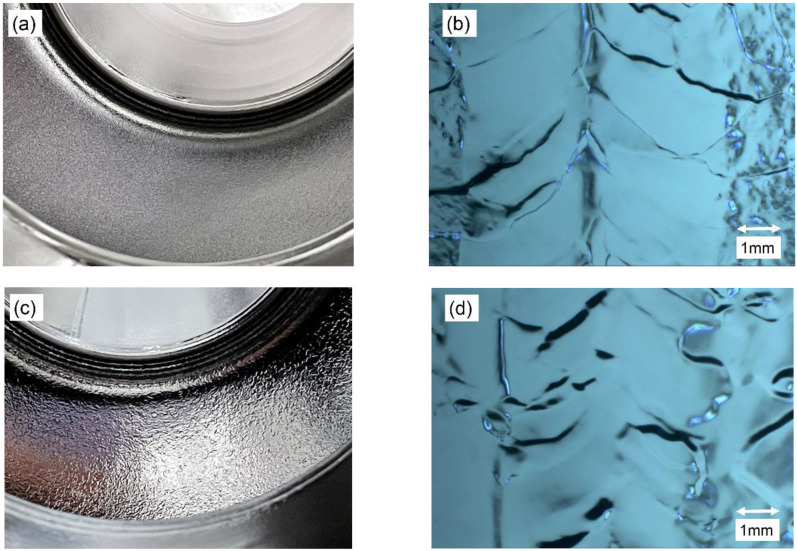
Images of the 650-MHz single-cell cavities that were EP processed. (**a**) Cell of 650S4, (**b**) equator of 650S4 using an inspection camera, (**c**) cell of 650S5, and (**d**) equator of 650S5 using an inspection camera.

After the EP process, the inner surfaces of the 650-MHz cavities were carefully examined by eye and with an inspection camera, which aimed to distinguish 650S4 from 650S5. The inspection camera is shown in Figure 7, and is similar to that of the High Energy Accelerator Research Organization (KEK) and Deutsches Elektronen Synchrotron (DESY) [30,31]. The high-resolution camera protruding from the cavity volume can move along the cavity axis, while the cavity can rotate automatically. Thus, every angle of cavity equator was photographed. Typical images of equator are shown in Figure 8b,d, and no obvious defects were found for both the 650-MHz single-cell cavities after the EP process. The equator of 650S5 was more vague than that of 650S4, which may have resulted from the lack of high-temperature annealing. The inner surfaces of the cells were almost the same for 650S4 and 650S5, and are shown in Figure 8a,c.

## 3. Vertical Test Results of 650-MHz Single-Cell Cavities after EP Process

**Figure 9:**
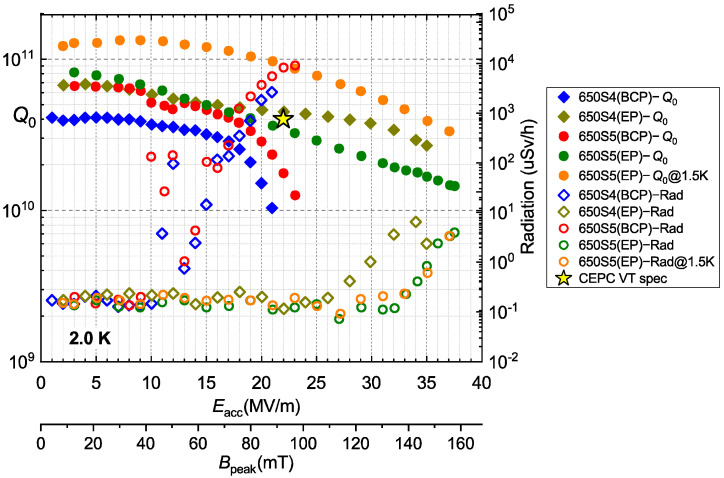
Vertical test results of 650-MHz single-cell cavities EP processed.

Afterwards, both the 650-MHz single-cell cavities received HPR, assembly with flanges, and low-temperature baking at 120 °C for 48 h. The vertical test results of the 650S4 and 650S5 after the EP process are shown in Figure 9, which demonstrates both the significantly higher *Q*_0_ and higher gradient than the BCP baseline.

Both 650S4 and 650S5 achieved an extremely high gradient, which quenched at 35.0 MV/m (*B*_peak_ = 147 mT) with *Q*_0_ of 2.7 × 10^10^ and 37.5 MV/m (*B*_peak_ = 158 mT) with *Q*_0_ of 1.4 × 10^10^, respectively. The values of *B*_peak_ achieved by 650S4 and 650S5 were remarkably high, which are close to the lower critical field (*H*_C1_, 170 mT) of niobium. Field emission was not serious during the vertical tests, which occurred above the gradient of 25 MV/m and was acceptable for such large SRF cavities. The *Q*_0_ of 650S4 was 4.5 × 10^10^ at 22.0 MV/m, which exceeded the CEPC VT spec. The *Q*_0_ of 650S5 was 3.4 × 10^10^ at 22.0 MV/m, which was a little lower than that of 650S4 above the gradient of 20 MV/m. The *Q*-slope phenomenon of 650S5 at 1.5 K was similar to that at 2.0 K. The relatively low *Q*_0_ of 650S5 in the medium and high fields may have resulted from the cancellation of high-temperature annealing, which can degas hydrogen in a cavity made of niobium. Hence, high-temperature annealing should be necessary for the achievement of high *Q*.

## 4. Discussion

Two 650-MHz single-cell fine-grain cavities (650S4 and 650S5) were fabricated for CEPC, which first underwent the BCP process. The *Q*_0_ and *E*_acc_ were not satisfactory during the vertical tests; hence, a new EP facility was developed for the 650-MHz cavity. Both 650S4 and 650S5 were subjected to different EP treatments in order to study the synergetic effect of BCP and EP processing. Approximately 40 μm of niobium material was removed from the inner surface of 650S4 via light EP. In contrast, the inner surface of 650S5 achieved a removal of 315 μm by bulk EP, which eliminated the effects of BCP and reset the surface.

After EP processing, *E*_acc_ and *Q*_0_ of both the 650-MHz cavities were significantly improved compared with the BCP baseline. 650S4 quenched at 35.0 MV/m with *Q*_0_ of 2.7 × 10^10^, with 650S5 at 37.5 MV/m with *Q*_0_ of 1.4 × 10^10^. Both 650S4 and 650S5 achieved state-of-the-art gradients for large elliptical cavities (frequency < 1 GHz). It seems that the recipe of 650S4 (bulk BCP + light EP) is as useful as that of 650S5 (bulk EP). BCP is easy and convenient, while EP is expensive and complicated. Therefore, the recipe of “bulk BCP + light EP” is attractive and efficient.

In addition, we attempted to omit high-temperature annealing and the following light EP, which was carried out for 650S5. This resulted in a slightly lower *Q*_0_ for 650S5 than that of 650S4. In the future, nitrogen doping and medium-temperature baking should be attempted to achieve high *Q* for 650-MHz single-cell cavities, which will also be extended to 650-MHz multi-cell cavities.

## Figures and Tables

**Table 1:** Main RF parameters of 650-MHz single-cell cavity.

Parameter	*B*_peak_/*E*_acc_ (mT/(MV/m))	*E*_peak_/*E*_acc_	*R*/*Q* (Ω)	*G* (Ω)
value	4.2	1.9	105	284

## Data Availability

Data sharing is not applicable.
